# Structure-odor relationships of linalool, linalyl acetate and their corresponding oxygenated derivatives

**DOI:** 10.3389/fchem.2015.00057

**Published:** 2015-10-06

**Authors:** Shaimaa A. Elsharif, Ashutosh Banerjee, Andrea Buettner

**Affiliations:** ^1^Department of Chemistry and Pharmacy, Food Chemistry, Emil Fischer Center, Friedrich-Alexander-Universität Erlangen-NürnbergErlangen, Germany; ^2^Department of Chemistry and Pharmacy, Pharmaceutical Chemistry, Emil Fischer Center, Friedrich-Alexander-Universität Erlangen-NürnbergErlangen, Germany; ^3^Department of Sensory Analytics, Fraunhofer Institute for Process Engineering and PackagingFreising, Germany

**Keywords:** Linalool, linalyl acetate, gas chromatography-olfactometry, odor threshold in air, 8-oxolinalyl acetate, 8-carboxylinalyl acetate, odor qualities, retention index

## Abstract

Linalool **1** is an odorant that is commonly perceived as having a pleasant odor, but is also known to elicit physiological effects such as inducing calmness and enhancing sleep. However, no comprehensive studies are at hand to show which structural features are responsible for these prominent effects. Therefore, a total of six oxygenated derivatives were synthesized from both **1** and linalyl acetate **2**, and were tested for their odor qualities and relative odor thresholds (OTs) in air. Linalool was found to be the most potent odorant among the investigated compounds, with an average OT of 3.2 ng/L, while the 8-hydroxylinalool derivative was the least odorous compound with an OT of 160 ng/L; 8-carboxylinalool was found to be odorless. The odorant 8-oxolinalyl acetate, which has very similar odor properties to linalool, was the most potent odorant besides linalool, exhibiting an OT of 5.9 ng/L. By comparison, 8-carboxylinalyl acetate had a similar OT (6.1 ng/L) as its corresponding 8-oxo derivative but exhibited divergent odor properties (*fatty, greasy, musty*). Overall, oxygenation on carbon 8 had a substantial effect on the aroma profiles of structural derivatives of linalool and linalyl acetate.

## Introduction

**Figure 1:**
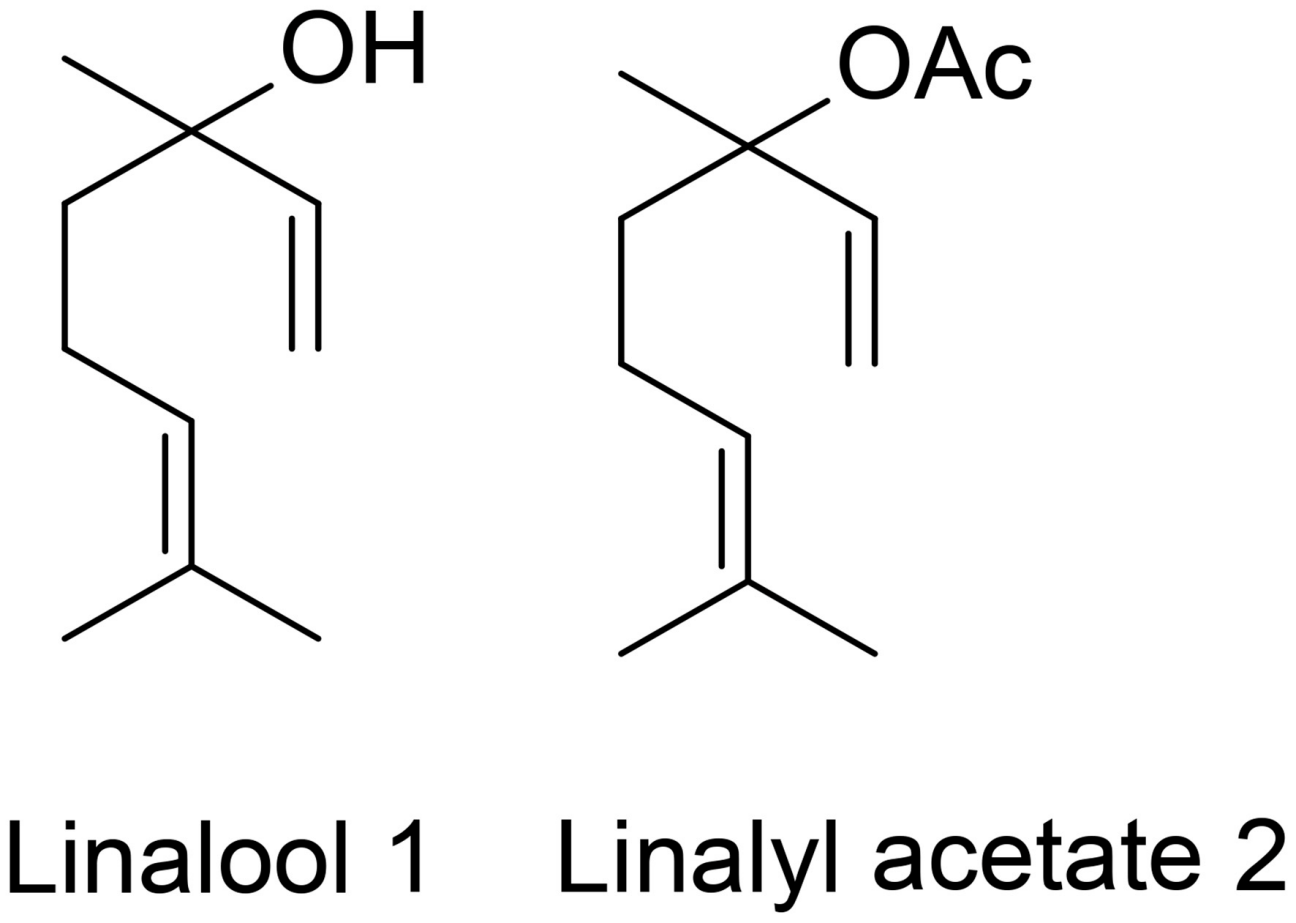
**Lavender oil main constituents**.

In folk medicines as well as aroma therapy, essential oils and fragrance compounds are being used as therapeutic agents for relieving pain, anxiety reduction and energy enhancement (Lahlou, 2004; Kako et al., 2008; Kiecolt-Glaser et al., 2008). Among them, due to their high volatility, the acyclic monoterpenes are a valuable class of compounds useful for the flavor and fragrance industries (King and Dickinson, 2003). One of the most important acyclic monoterpene substances is linalool **1** which represents about 70% of the terpenoids of floral scents (Stashenko and Martinez, 2008). In perfumery, linalool is a commonly used fragrant ingredient being a component of many perfumes top notes and being found in 60–90% of cosmetic products (Cal and Krzyzaniak, 2006). Its odor is described in literature as floral, citric, fresh and sweet (d'Acampora Zellner et al., 2007). It is also added to household cleaning agents, furniture care products, waxes, as well as to processed food and beverages, as a fragrance and flavor agent. Linalool is found in the essential oils of over 200 plant species, belonging to different families (Stashenko and Martinez, 2008). For example, linalool and its ester form, linalyl acetate **2**, are the lavender oil main constituents (Figure 1; Buchbauer et al., 1991). The odor of linalyl acetate is described as floral, sweet and citric, and additionally as minty and slightly caraway-like (d'Acampora Zellner et al., 2007).

**Scheme 1:**
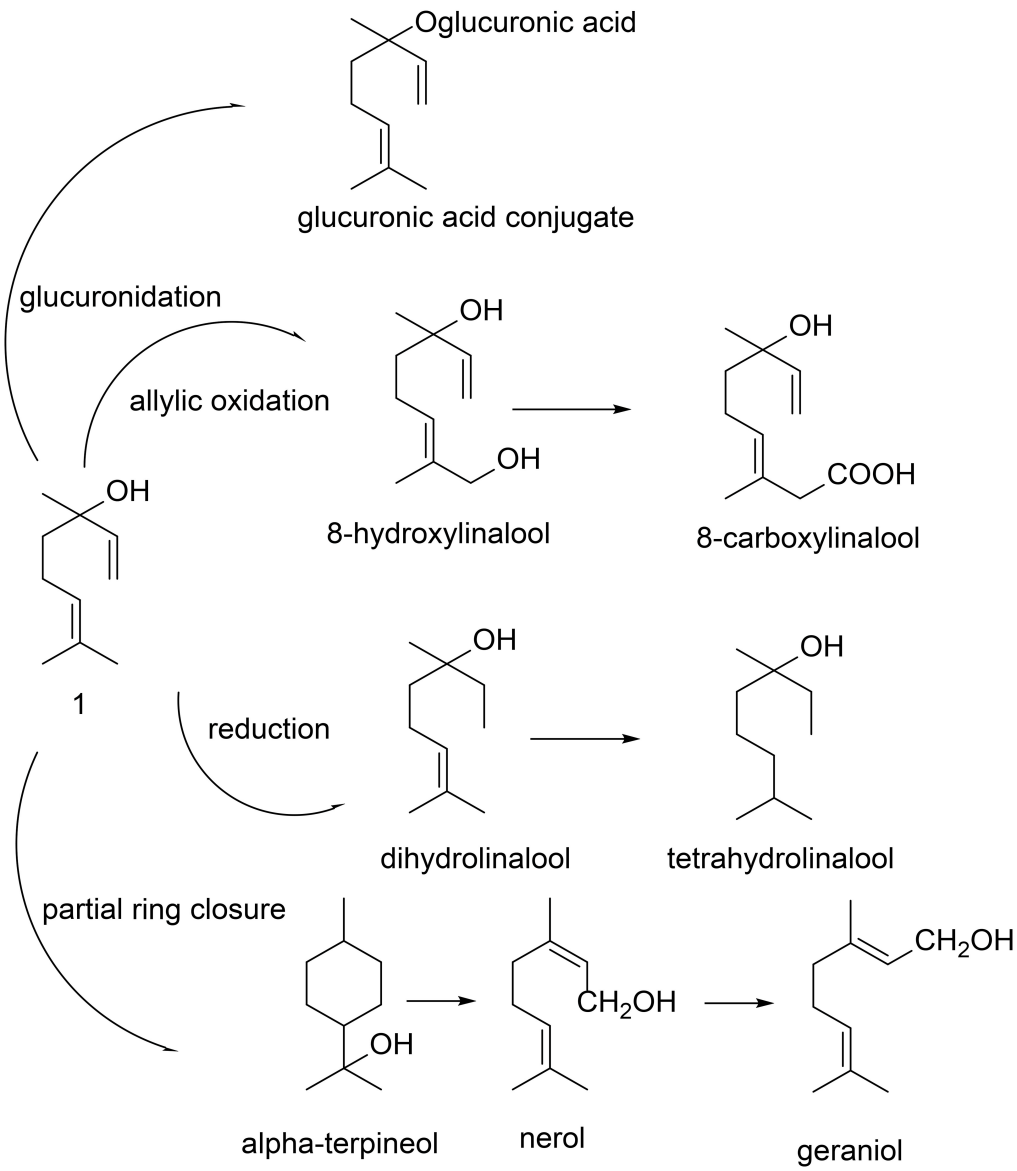
**Main linalool metabolic pathway in mammals (scheme modified from Aprotosoaie et al., 2014)**.

Lavender oils are widely used to enhance sleep. Thereby, it has been demonstrated that lavender aromatics can improve sleep in the elderly (Hudson, 1996) and infants (Field et al., 2008). Furthermore, exposure to lavender odors during sleep results in increased duration of deep slow-wave stage sleep (Goel et al., 2005). A closely related therapeutic effect is anxiety reduction, having also been reported for lavender essential oil usage (Tasev et al., 1969). In view of this, linalool has been demonstrated to not only activate olfactory receptors but also to modulate ion channel receptor potentials such as the transient receptor potential channels (TRP) and to potentiate γ-aminobutanoic acid receptor A (GABA_A_)-receptor response in the central nervous system (Kessler et al., 2012, 2014); the latter receptor system has been shown to be strongly involved in sedative, anxiolytic and calming processes. TRP channels, on the other hand, are involved in numerous physiological conditions and diseases, and their potential modulation by aroma compounds such as linalool is discussed comprehensively in Friedland and Harteneck (2015). Based on these observations there is a general understanding that linalool plays a valid role in the calming response in humans. However, metabolic side products of linalool, both in plants as well as animals or humans have not been regarded comprehensively in view of either smell or other physiological effects. For gaining deeper insights into the metabolic origin and further fate of linalool and its derivatives, studies were carried out to investigate the metabolism of these substances both in plants (Luan et al., 2006) and animals (Chadha and Madyastha, 1984). Experimental studies on rats using ^14^C-labeled linalool showed that it is rapidly absorbed from the intestinal tract after oral administration. The major part of linalool is metabolized by the liver to polar compounds which are mainly excreted in urine as free form or conjugates; only minor amounts are excreted via the feces. Allylic oxidation becomes an important pathway upon repeated administration, being mediated by the cytochrome P-450 system. 8-Hydroxylinalool and 8-carboxylinalool were detected as major metabolites after 20 days administration of linalool in rats. A minor part undergoes partial ring closure to α-terpineol, with the generation of small amounts of geraniol and nerol. These metabolites are also excreted in urine as free forms or conjugates. Products of linalool reduction (dihydro-, tetrahydrolinalool) were also identified in rodent urine (Aprotosoaie et al., 2014). A significant proportion of orally administered linalool follows intermediary metabolic pathways as shown in Scheme 1 (scheme modified from Aprotosoaie et al., 2014).

8-Hydroxylinalool was not only found as a metabolite in mammalian species, but also as an oxidation product isolated from the grape berry mesocarp after linalool was applied to it (Luan et al., 2006). 8-Carboxylinalool was found to be among the constituents of the fruits of *Euterpe oleracea* (Chin et al., 2008) and the flower of *Albizia julibrissin* (Yahagi et al., 2012). Linalyl acetate metabolism was also studied in *Pseudomonas incognita* (Renganathan and Madyastha, 1983), where it was shown that the C-8-methyl moiety is subjected to selective oxidation, giving 8-hydroxylinalyl acetate which is then oxidized to 8-oxo and 8-carboxylinalyl acetate, respectively. Apart from that, 8-oxolinalyl acetate was first isolated from lavandin oil and hence reported as a constituent of a natural product (Mookherjee and Trenkle, 1973). 8-Carboxylinalyl acetate was found in trace amounts (<0.01%) in Jabara (*Citrus jabara* Hort ex. Tanaka) peel extract (Mookherjee and Trenkle, 1973; Table 1).

**Table 1:** **Retention indices and occurrence of linalool and its derivatives**.

**Entry**	**Odorant**	**RI^a^**		**Previously identified in**
		**DB5**	**FFAP**	
1	Linalool	1108	1550	Some examples: Wood of *Aniba rosaeodora Ducke*, Lauraceae^b^, Flowering tops of *Lavandula officinalis, L. angustifolia Mill*., Lamiaceae^c^, *Coriandrum sativum* L., Apiaceae^d^, Flowers of Citrus *sinensis Osbeck*, Rutaceae^e^
2	Linalyl acetate	1264	1563	*Lavandula angustifolia Miller^f^, Micromeria kerneri* and *Micromeria juliana* Lamiaceae^g^, *Origanum vulgare* Lamiaceae^h^.
3	8-Oxolinalool	1350	2150	*Narcissus trevithian* and *Narcissus geranium* Amaryllidaceae^i^
4	8-Oxolinalyl acetate	1490	2133	Lavandin oil^j^
5	8-Hydroxylinalyl acetate	1530	2333	As a linalyl acetate metabolite by *pseudomonas incognita*^k^
6	8-Hydroxylinalool	1380	2320	*Chamaecyparis Obtuse*^l^*, Vitis vinifera (muscat grape skins)*^*m*^*, Pluchea indica*^n^, As a linalool metabolite in urine of rats^o^, and as a linalool oxidation product in grape berry mesocarp^p^.
7	8-Carboxylinalool	1540	1929	Fruits of *Euterpe oleracea*^q^, flower of *Albizia julibrissin*^r^.
8	8-Carboxylinalyl acetate	1650	1957	Trace amounts in *Jabara (Citrus jabara Hort* ex. Tanaka)^s^

a *retention indices were determined as described by Van Den Dool and Kratz (1963)*.

b *Chantraine et al. (2009)*.

c *Ozek et al. (2010)*.

d *Tsagkli et al. (2012)*.

e *Miguel et al. (2008)*.

f *Buchbauer et al. (1991)*.

g *Kremer et al. (2014)*.

h *Andi et al. (2012)*.

i *Van Dort et al. (1993)*.

j *Mookherjee and Trenkle (1973)*.

k *Renganathan and Madyastha (1983)*.

l *Matsubara et al. (1990)*.

m *Strauss et al. (1988)*.

n *Uchiyama et al. (1989)*.

o *Chadha and Madyastha (1984)*.

p *Luan et al. (2006)*.

q *Chin et al. (2008)*.

r *Yahagi et al. (2012)*.

s *Omori et al. (2011)*.

Therefore, we conclude that the carbonyl, the hydroxyl and the carboxylic acid functional groups in α-position to the double bond are very common in nature. These metabolites have been previously synthesized as regio-selectively deuterated compounds for the investigation of their bioconversion into lilac during an *in vivo* feeding experiment to *Syringa vulgaris* L., Oleaceae, to study the metabolic pathway of linalool and its derivatives (Kreck et al., 2003). Non-deuterated derivatives were used as reference substances for elucidation of compounds in essential oils isolated from plants to reveal their structural and organoleptic properties (Van Dort et al., 1993). However, the latter study does not contain any explanation of accurate methods of smell determination, nor discuss any further potential physiological impact on humans. Accordingly, neither the odor qualities and odor thresholds of these substances are investigated systematically, nor is it clear what makes linalool so unique for its odor but also other physiological effects.

Based on these considerations we synthesized, starting from **1** and **2**, previously reported metabolites and hypothetical derivatives of linalool and its related ester in order to determine their respective odor qualities and thresholds. We thereby aimed at elucidating if linalool itself represents the most potent and characteristic member of this substance group or if any other potent compounds are promising natural physiological chemo-stimuli in humans. Finally, the aim was to provide a substance library that should further aid in future analytical studies, with compiled data on Retention Indices (RI-values) as well as mass spectrometric and nuclear magnetic resonance data.

## Materials and methods

### Chemicals

The following chemicals were purchased from the suppliers given in parentheses**:** linalool, linalyl acetate, selenium dioxide, sodium borohydride, methanol, methanol anhydrous, ethanol, dioxane, tert-butyl alcohol, 2-methyl-2-butene, petroleum ether, sodium chlorite, sodium dihydrogen phosphate, ethyl acetate, hexane, magnesium sulfate (Aldrich, Steinheim, Germany), diethyl ether (Fisher Scientific, Loughborough).

### General methods

All reactions requiring anhydrous conditions were carried out under nitrogen and the solvents were dried before use to remove moisture using appropriate drying solvents. All reactions were monitored by TLC using Kieselgel 60 F_254_ plates. Visualization of the reaction components was achieved using UV fluorescence (254 nm) and KMnO_4_ stain. Column chromatography was carried out over silica gel 60. The yields reported are after purification.^1^H and ^13^C NMR spectra were recorded in deuterated solvents and chemical shifts (δ) are quoted in parts per million (ppm) calibrated to TMS (^1^H and ^13^C). Coupling constants (*J*) were measured in Hertz (Hz). The following abbreviations are used to describe multiplicities: s = singlet, d = doublet, t = triplet, q = quartet, b = broad, m = multiplet. The identity of all intermediates and synthetic products was determined by MS/EI.

### Nuclear magnetic resonance (NMR) spectra

^1^H and ^13^C NMR spectra were recorded in CDCl_3_ on an Avance 360 spectrometer, 360 MHz, and Avance 600, 600 MHz (Bruker Biospin, Rheinstetten, Germany) at room temperature operated at 360 or 600 MHz (^1^H) and 90 or 150 MHz (^13^C), with tetramethylsilane (TMS) as internal standard.

### Gas chromatography-olfactometry (GC/O) and GC-electron impact-mass spectrometry (GC-EI-MS)

GC-O analyses were performed with a Trace GC Ultra (Thermo Fisher Scientific GmbH, Dreieich, Germany) by using the following capillaries: FFAP (30 m × 0.32 mm fused silica capillary, free fatty acid phase FFAP, 0.25 μm; Chrompack, Mühlheim, Germany) and DB5 (30 m × 0.32 mm fused silica capillary DB-5, 0.25 μm; J&W Scientific, Fisons Instruments). The helium carrier gas flow was set at 2.0 mL/min. The compounds eluting at the end of the capillaries were split with a Y-splitter (J&W Scientific; ratio 1:1 v/v) and transferred via two deactivated capillaries (0.5 m × 0.2 mm, J &W Scientific) to a flame ionization detector and a heated sniffing port (temperature: 250°C). The samples were applied onto the capillary using a cold-on-column injector at 40°C. After 2 min, the oven was heated at a rate of 15°C/min to 240°C and held for 2 min. GC-EI MS analyses were performed with an Agilent MSD 5975C (Agilent Technologies, Waldbronn, Germany) and a Thermo ITQ 900 (Thermo Fisher Scientific, Dreieich, Germany) with the capillaries described above. Mass spectra in the electron impact mode (EI-MS) were generated at 70 eV.

### Retention indices (RI)

Retention indices (Table 1) were determined by the method previously described by Van Den Dool and Kratz (1963).

### Evaluation of odor quality

The odor qualities were determined during GC-O evaluation by the aid of panelists who were trained volunteers from the University of Erlangen (Erlangen, Germany), exhibiting no known illness at the time of examination and with audited olfactory function. In preceding weekly training sessions the assessors were trained for at least half a year in recognizing orthonasally about 90 selected known odorants at different concentrations according to their odor qualities and in naming these according to an in-house developed flavor language.

### Determination of odor thresholds

Odor thresholds were determined in air following the procedure described by Czerny et al. (2011) using (*E*)-dec-2-enal as an internal odor standard. This procedure offers the advantage that compounds that might be present as odor-active impurities in the reference compound are separated from the target odorants during the chromatographic separation step. In consequence, an influence of such components on the results is avoided. Also, odor thresholds can be compared to each other on an absolute basis without interference with any matrix system as would be the case e.g., when determining odor thresholds in water. The detection odor thresholds of the panel were calculated as the geometric mean of the individual thresholds according to Czerny et al. (2008).

### Synthesis, general procedures

#### General procedure 1(GP1)

**Scheme 2:**
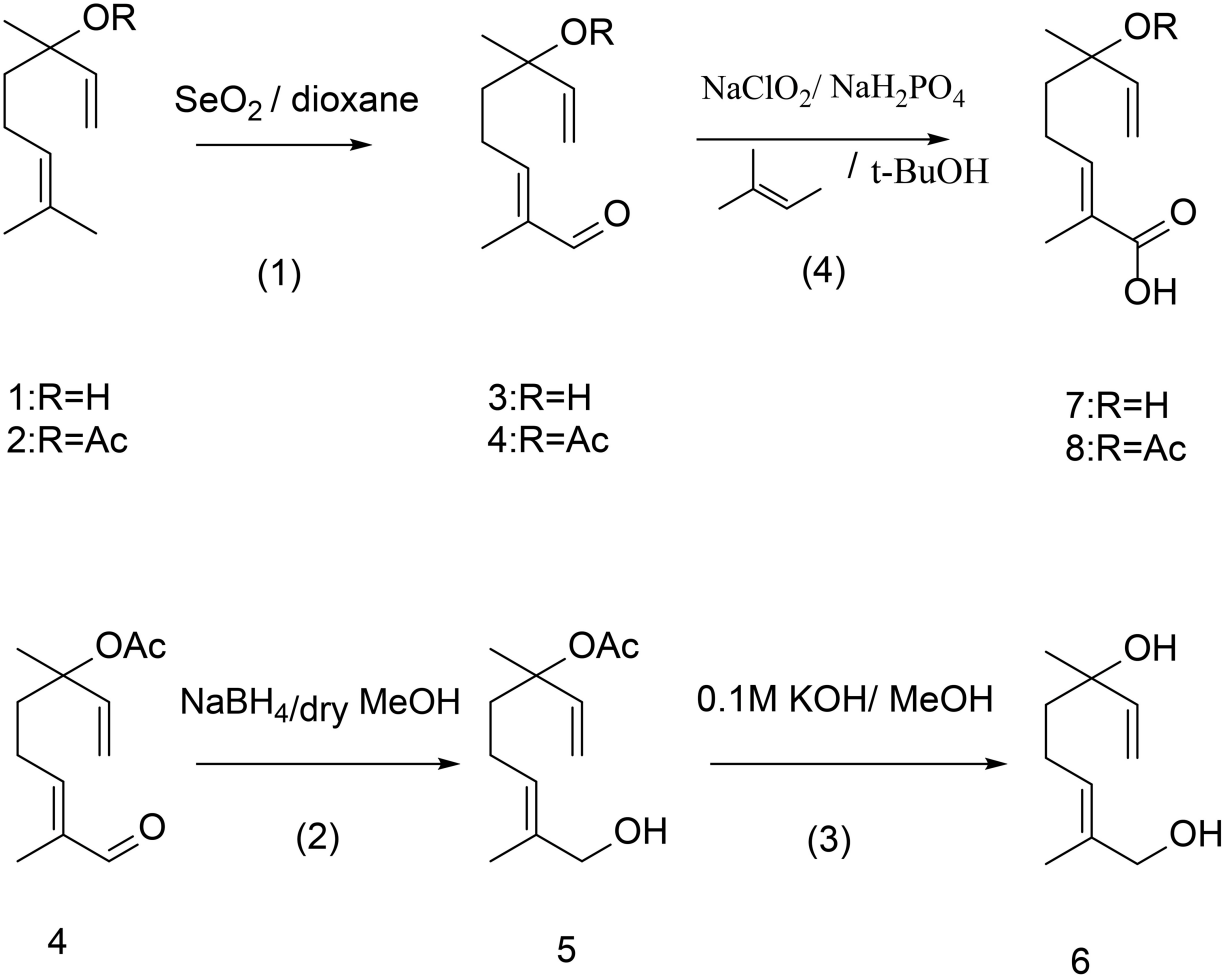
**Synthetic pathways for the synthesis of linalool and linalyl acetate oxygenated derivatives following procedures 1-4**.

Generally, the method of Wakayama et al. (1973) was used (Scheme 2). Compounds **1** and **2** and selenium dioxide (1 eq.) were dissolved in dioxane/ethanol 9:1 (v/v), and the solution was heated at 80°C for 5 h. After removal of selenium deposit by filtration, the solvent was removed under reduced pressure using a rotary evaporator. The residue was treated with diethyl ether/petroleum ether 1:1 (v/v), and after removal of the solvent, the residue was purified by flash chromatography on silica gel 60 (Merck), with a mobile phase of petroleum ether/diethyl ether, affording the crude compounds **3** and **4**, respectively.

#### (E)-3,7-dimethyl-8-oxoocta-1,6-dien-3-ol (3), *8-oxolinalool*

Following GP1, from **1** (4.8 g, 31.1 mmol) and selenium dioxide (3.4 g, 30.4 mmol) in 30 ml dioxane/ethanol 9:1 (v/v), compound **3** was prepared. Flash chromatographic purification with petroleum ether/diethyl ether 1:4 (v/v) yielded 1.4 g (29%) of **3** as orange oil.^1^H NMR (600 MHz, *CHLOROFORM-d*) δ ppm 9.38 (1 H, s), 6.42–6.56 (1 H, m), 5.92 (1 H, dd, *J* = 17.26, 10.67 Hz), 5.25 (1 H, dd, *J* = 17.26, 0.91 Hz), 5.11 (1 H, dd, *J* = 10.90, 0.91 Hz), 2.35–2.45 (2 H, m), 1.74 (3 H, s), 1.61–1.71 (2 H, m), 1.31–1.35 (3 H, m).^13^C NMR (91 MHz, *CHLOROFORM-d*) δ ppm 195.2., 154.6, 144.3, 139.2, 112.4, 72.9, 40.3, 28.1, 23.8, 9.1.MS (EI) *m/z* (%) (rel.int.): 168 [M^+^] (1), 98(15), 87(27), 82(24), 71(100), 55(33), 43(58), 41(23).

#### (E)-3,7-dimethyl-8-oxoocta-1,6-dien-3-yl-acetate (4), *8-oxolinalyl acetate*

Following GP1, from **2** (5 g, 25 mmol) and selenium dioxide (2.7 g, 25 mmol) in 15 ml dioxane/ethanol 9:1 (v/v), compound **4** was prepared. Flash chromatographic purification with petroleum ether/diethyl ether 3:2(v/v) yielded 1.4 g (29%) of **4** as orange oil.^1^H NMR (600 MHz, *CHLOROFORM-d*) δ ppm 9.39 (1 H, s), 6.44–6.50 (1 H, m), 5.96 (1 H, dd, *J* = 17.56, 11.14 Hz), 5.15–5.26 (2 H, m), 2.37 (2 H, q, *J* = 7.93 Hz), 2.06–2.12 (1 H, m), 2.02 (3 H, s), 1.87–1.95 (1 H, m), 1.74 (3 H, s), 1.59 (3 H, s).^13^C NMR (151 MHz, *CHLOROFORM-d*)δ ppm 195.1, 169.9, 153.7, 141.1, 139.5, 113.8, 82.3, 38.1, 23.79, 23.79, 22.1, 9.12. MS (EI) *m/z* (%) (rel.int.): 210 [M^+^] (1), 150(18.38), 135(14), 121(19), 107(18.05), 93(26), 82(41), 71(46), 55(29), 43(100).

### Procedure 2

#### (E)-8-hydroxy-3,7-dimethylocta-1,6-dien-3-yl-acetate (5), 8-hydroxylinalyl acetate

Compound **4** (800 mg, 3.81 mmol) was dissolved in dry methanol (40 ml) and sodium borohydride (NaBH_4_; 1.8 g, 4.72 mmol) was added (Liu et al., 2003; Scheme 2). The solution was allowed to stir at −10°C. After 1 h, water was added and the reaction mixture was extracted with dichloromethane (DCM). The organic layer was dried over sodium sulfate. After removal of the solvent, the residue was subjected to flash chromatography eluted with petroleum ether/diethyl ether 2:3 (v/v) and yielded 626 mg (77%) of **5** as light yellow oil.^1^H NMR (360 MHz, *CHLOROFORM-d*) δ ppm 5.97 (1 H, dd, *J* = 17.48, 10.90 Hz), 5.36–5.43 (1 H, m), 5.15 (2 H, dd, *J* = 17.48, 11.13 Hz), 3.99 (2 H, d, *J* = 5.45 Hz), 2.03–2.09 (2 H, m), 2.01 (3 H, s), 1.75–1.96 (2 H, m), 1.66 (3 H, s), 1.55 (3 H, s). ^13^C NMR (91 MHz, *CHLOROFORM-d*) δ ppm 169.9, 141.7, 135.2, 125.4, 113.3, 82.8, 68.8, 39.4, 23.7, 22.2, 21.9, 13.6. MS (EI) *m/z* (%) (rel.int.): 211 [M^+^-1] (1), 134(7), 119(27), 93(46), 79(35), 67(30), 55(24), 43(100).

### Procedure 3

#### (E)-2, 6-dimethylocta-2,7-diene-1,6-diol (6), 8-hydroxylinalool

Compound **5** (311 mg, 1.46 mmol) was dissolved in methanol (50 ml) and 0.1 M KOH (50 ml) was added (Hasegawa, 1983; Scheme 2). The reaction mixture was allowed to stir at 60°C. After 4 h, the solution was extracted with DCM and the organic layer was dried over sodium sulfate. The solvent was removed under reduced pressure and flash chromatographic purification with petroleum ether/diethyl ether 1:4 (v/v) yielded 203.8 mg (82%) of **6** as transparent oil.^1^H NMR (600 MHz, CHLOROFORM-d) δ ppm 5.93 (1 H, dd, *J* = 17.19, 10.76 Hz), 5.41–5.45 (1 H, m), 5.16 (2 H, dd, *J* = 17.75, 10.95 Hz), 4.00 (2 H, s), 2.03–2.16 (2 H, m), 1.68 (3 H, s), 1.59–1.65 (2 H, m), 1.31 (3 H, s). ^13^C NMR (151 MHz, *CHLOROFORM-d*) δ ppm 144.9, 135.0, 125.9, 111.8, 73.3, 68.9, 41.7, 27.9, 22.3, 13.6. MS (EI) *m/z* (%) (rel.int.): 170 [M^+^] (1), 150(16), 135(13), 131(18), 107(17), 95(25), 82(39), 71(44), 55(28), 43(100).

### General procedure 4 (GP4)

Pinnick oxidation was used for the following syntheses (Pinnick et al., 1981; Scheme 2). The aldehydes **3** and **4** were dissolved in 25 ml of tert-butyl alcohol and 6 ml 2-methyl-2-butene. A solution of sodium chlorite (9.2 eq.) and sodium dihydrogenphosphate (6.9 eq.) in 10 ml water was added drop wise over a 10 min period. The reaction mixture was stirred at room temperature overnight. Volatile components were then removed under vacuum, the residue was dissolved in 30 ml water and this was extracted with two 15 ml portions of hexane. The aqueous layer was acidified to pH 3 with HCl and extracted with three 20 ml portions of ether. The combined ether layers were washed with 50 ml cold water dried and concentrated to give **7** and **8**, respectively.

### (E)-6-hydroxy-2,6-dimethylocta-2,7-dienoic-acid (7), 8-carboxylinalool

Following GP4, Compound **3** (800 mg, 4.75 mmol) was dissolved in 25 ml tert-butyl alcohol and 6 ml 2-methyl-2-butene. A solution of sodium chlorite (3.95 gm, 43.7 mmol) and sodium dihydrogenphosphate (3.93 gm, 32.7 mmol) in 10 ml water was added dropwise over a 10 min period, compound **7** was prepared. Flash chromatographic purification with ethyl acetate/methanol 9.5:0.5 (v/v) yielded 373.7 mg (42.6%) of **7** as white solid specks.^1^H NMR (600 MHz, CHLOROFORM-d) δ ppm 6.76–6.94 (1 H, m), 5.89 (1 H, dd, *J* = 17.37, 10.58 Hz), 5.22 (1 H, dd, *J* = 17.37, 1.13 Hz), 5.08 (1 H, dd, *J* = 10.76, 0.94 Hz), 2.14–2.33 (2 H, m), 1.81 (3 H, s), 1.64 (2 H, m, *J* = 18.70, 10.60 Hz), 1.30 (3 H, s). ^13^C NMR (151 MHz, *CHLOROFORM-d*) δ ppm 172.1, 144.5, 144.4, 127.1, 112.2, 72.9, 40.4, 27.9, 23.6, 12.0. MS (EI) *m/z* 182 [M^+^-2] (1), 151(4), 138(7), 121(15), 111(14), 103(16), 95(16), 82(11), 71(100), 67(18), 55(24).

### (E)-6-acetoxy-2,6-dimethylocta-2,7-dienoic-acid (8), *8-carboxylinalyl acetate*

Following GP4, compound **4** (0.3 gm, 1.24 mmol) was dissolved in 25 ml tert-butyl alcohol and 6 ml 2-methyl-2-butene. A solution of sodium chlorite (1.08 gm, 11.4 mmol) and sodium dihydrogenphosphate (1.05 gm, 8.55 mmol) in 10 ml water was added dropwise over a 10 min period, compound **8** was prepared. Flash chromatographic purification with ethyl acetate/methanol 9.5:0.5 (v/v) yielded 131.8 mg (47%) of **8** as light yellow oil.^1^H NMR (600 MHz, *CHLOROFORM-d*) δ ppm 6.87–6.92 (1 H, m), 5.95 (1 H, dd, *J* = 17.37, 10.95 Hz), 5.17 (2 H, dd, *J* = 17.37, 10.95 Hz), 2.17–2.24 (2 H, m), 2.02 (3 H, s), 1.84–2.00 (2 H, m), 1.83 (3 H, s), 1.57 (3 H, s). ^13^C NMR (91 MHz, CHLOROFORM-d) δ ppm 173.1, 169.8, 144.1, 141.1, 127.2, 113.5, 82.3, 38.1, 23.6, 23.3, 22.0, 11.8. MS (EI) *m/z* 226 [M^+^] (2), 166(15), 148(19), 121(100), 105(84), 91(91), 79(98), 67(89), 55(42).

## Results

Odor qualities for **1** and **2** and their C-8 oxygenated synthesized derivatives were investigated by trained panelists using GC-O, the obtained attributes are shown in Table 2. It was found that linalool, 8-oxolinalool and 8-hydroxylinalool exert the same or at least closely related odor qualities. Odor attributes named by the panelists were *citrus-like, sweet, soapy*, and *lemon-like*, whereas no odor was perceived at the sniffing port in case of 8-carboxylinalool. It is worth mentioning that the latter compound was odorless for all panelists in the concentration levels evaluated, that means at a concentration up to about 200 μg/ml.

**Table 2:** **Odor qualities of all eight panelists (P1 to P8) and median of the odor threshold of all compounds**.

**Entry^a^**	**Structure**	**Odor qualities^b^**								**OT [ng/L(air)]**	
		**P1**	**P2**	**P3**	**P4**	**P5**	**P6**	**P7**	**P8**	**Range**	**Median**
1	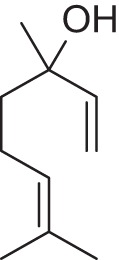	Citrus, soapy, fresh	Citrus, soapy, lemon-like	Flowery, balsamic	Citrus, sweet	Citrus, flower	Citrus, soapy, flower	Lemon-like, green, fatty	Citrus, flowery	2.1–8.4	2.1
2	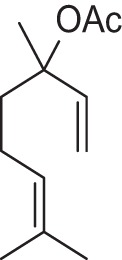	Citrus, fatty, sweet	Citrus, fatty	Citrus, fresh, acidic	Sweet, fatty	Citrus	Soapy, fatty	Lemon-like, Melissa	Citrus	12.7–407	152.5
3	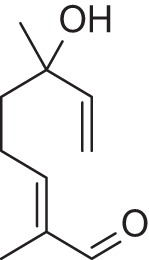	Fatty	Lemon-like, sweet, citrus, soapy	Fatty, fruity, balsamic	Fatty, citrus	Lemon-like, citrus	Fatty, citrus	Fresh, citrus, lemon-like	Fatty, citrus, soapy	4.8–305	28.55
4	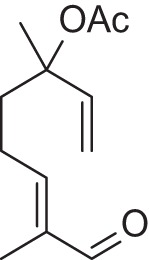	Citrus, fatty, soapy	Lemon-like, fatty, sweet	Citrus, fatty, linalool-like, soapy, balsamic	Sweet, fatty	Lemon-like, flower	Soapy, fatty	Lemon-like, green, fresh	Citrus, fatty	0.6–79	4.9
5	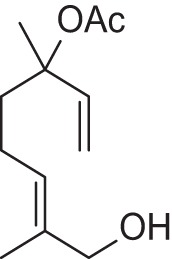	Citrus, fatty	Citrus, soapy	Citrus, orange, flowery, balsamic	Citrus, fatty, fresh, fruity	Fresh, fruity	Citrus, fatty	Lemon-like, fresh	Citrus, flowery	4.9–634	198
6	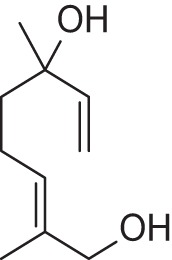	Citrus	Lemon-like, sweet, flowery	Citrus, fresh	Citrus, sweet	Fresh	Citrus, soapy, sweet	Lemon-like, orange	Citrus	7.7–989	247
7	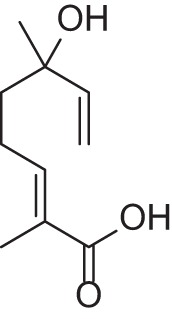	Odorless	Odorless	Odorless	Odorless	Odorless	Odorless	Odorless	Odorless	–	–
8	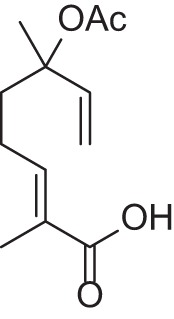	Fatty, musty, rancid	Sweet, musty	Fatty, greasy	Fatty, greasy	Fatty	Fatty	Fatty	Fatty, waxy	1.5–24	6.1

a *Numbering refer to Table 1*.

b *Odor qualities as perceived at the sniffing port*.

The odor of linalyl acetate and its derivatives was described as *citrus-like, soapy, fatty*, and *fresh* similarly to linalool with the sole exception of 8-carboxylinalyl acetate; the smell of this substance was described as *waxy, fatty, musty, rancid*, and *greasy*. Panelists did not mention in any case the attributes *citrus-like, soapy, fresh*, or *lemon-like* for the latter compound.

As shown in Table 2, it is worth mentioning that the attributes provided by 60–70% of the panelists in case of linalool were *citrus-like* and *flowery*, whereas *soapy* was selected by only 37% of the panel as descriptor. Only one panelist described linalool odor as *balsamic*. Regarding the 8-oxolinalool odor, all panelists agreed on the attributes *fatty* and *citrus-like*, while 25% of the panel named the attribute *soapy*. In contrast to the latter two compounds, exhibiting intense odor, 8-hydroxylinalool was perceived by most panelists as low in odor intensity even in a concentration of 390 μg/ml at the sniffing port. About 88% of the panelists provided the attribute *citrus-like* for description of the latter compound, while only one panelist perceived the substance as *soapy* and *flowery*; unlike for linalool and the 8-oxolinalool, no panelist mentioned *fatty* as a descriptor for 8-hydroxylinalool.

In view of linalyl acetate, the *citrus-like* impression was reported by 60% of the panelists; *fatty* and *soapy* were named by 25% while *lemon-like* and *melissa-like* attributes were given by only one panelist. Furthermore, for the 8-oxolinalyl acetate odor 75% of the panelists concordantly reported a *fatty* attribute. About 40% of the panel further described the odor as *lemon-like* and *citrus-like* while only one panelist gave the attribute *balsamic* and *linalool-like*. Nevertheless, the main 8-hydroxylinalyl acetate odor attributes were *citrus-like*, being named by most of the panelists (70–80%), while *fresh* and *fruity* were given by only 20% of the panel. In contrast to all the previously mentioned odors, the 8-carboxylinalyl acetate smell was described as *fatty* and additionally as *musty, rancid* and *greasy*, but not as *soapy*.

When evaluating more closely the individual odor threshold results of the panelists, it becomes evident that there are some inter-individual differences that do not only vary from a compound to another but also for a specific substance. Overall, all compounds were perceived with an intense to medium intense odor with the sole exception of 8-hydroxylinalool which imparted weak odor intensity. Thereby, one panelist was exceptionally sensitive to all compounds, recording a threshold value as low as 0.6 ng/L for the 8-oxolinalyl acetate, thus, being the lowest threshold value determined within this study (Table 3). In case of linalool, 60% of the panelists achieved a threshold value of 2.1 ng/L while in case of the other compounds not more than two panelists concordantly displayed the same odor threshold. To name but one example, the highest threshold was recorded for two panelists for 8-hydroxylinalool with 989 ng/L; this value is by a factor of 128 higher than the lowest recorded threshold (7.7 ng/L), in this case again achieved by the sensitive panelist who was discussed before.

**Table 3:** **Odor thresholds OT (GC-O) of all eight panelists (P1 to P8) of all compounds**.

**Entry^a^**	**Odorant**	**OT [ng/L(air)]^b^**								**Group^c^**	**Literature^d^**
		**P1**	**P2**	**P3**	**P4**	**P5**	**P6**	**P7**	**P8**		
1	Linalool	8.4	8.4	2.1	2.1	2.1	2.1	4.2	2.1	3.2	n.r.
2	Linalyl acetate	407	203	51	102	12.7	203	407	51	110.9	n.r.
3	8-Oxolinalool	305	38	19	76	4.8	9.5	305	19.1	38.1	n.r.
4	8-Oxolinalyl acetate	2.8	4.9	4.9	79	0.6	4.9	10	10	5.9	n.r.
5	8-Hydroxylinalyl acetate	634	634	40	79	4.9	317	317	20	102.8	n.r.
6	8-Hydroxylinalool	989	989	247	247	7.7	31	494	62	160.3	n.r.
7	8-Carboxylinalool	–	–	–	–	–	–	–	–	–	n.r.
8	8-Carboxylinalyl acetate	3.1	3.1	6.1	6.1	1.5	24	24	6.1	6.1	n.r.

a *Numbering refer to Table 1*.

b *Odor thresholds in air were determined as described by Ullrich and Grosch (1987)*.

c *Group odor threshold was calculated as a geometric mean of the individual thresholds of panelists*.

d *n.r.: Odor threshold was not reported previously*.

When comparing linalool with its oxygen-containing analogs, we found that linalool is the most potent odorant having an odor threshold of 3.2 ng/L in air (Table 3). All other compounds investigated within this study exhibited an odor threshold of at least a factor of 2 higher than the threshold of linalool. To analyze the secret beyond this intensive odor, it is feasible to have a closer look at the respective substituents on the monoterpene structure.

**Figure 2:**
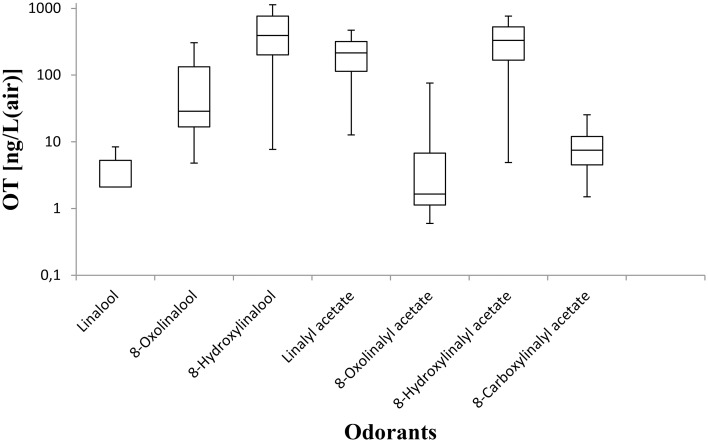
**Influence of oxygenated functional groups on the odor threshold of odorants**.

Addition of an aldehyde group at C-8 of linalool increases the threshold by a factor of 12 (38.1 ng/L). Reduction of this aldehyde to the corresponding alcohol, giving the 8-hydroxylinalool, results in a dramatic decrease in the potency, and a large increase in the threshold value (160.31 ng/L). Upon oxidation of the 8-oxolinalool to the corresponding 8-carboxylinalool the odor totally disappears. This means that the C-3 hydroxy group is the only substituent responsible for the linalool high potency and low threshold value, whereas any substituents on C-8, referring to this study (see Figure 2), especially the aldehydic or the alcoholic functional groups, can still own the same linalool pleasant smell but lack its potency.

For linalyl acetate, the acetate ester of linalool, the odor threshold was determined to be 110.9 ng/L, which is the highest value in relation to linalool despite its sweet, citrus fresh odor. Surprisingly, 8-oxolinalyl acetate, the linalyl acetate-8-aldehyde, was found to be the most potent compound of its corresponding ester derivatives (see Figure 2) with an odor threshold of 5.9 ng/L which is close to the odor threshold of linalool itself. Its odor quality was also described to be *linalool-like* and very intense compared to that of its parent substance, the linalyl acetate. Again, the reduction of the C-8 aldehyde to the respective alcohol gives the 8-hydroxylinalyl acetate with an odor threshold of 102.8 ng/L which is comparatively lower than that of linalyl acetate itself. Interestingly, the 8-carboxylinalyl acetate, the oxidation product of the 8-oxolinalyl acetate, retained the odor threshold (6.1 ng/L) to be nearly the same as for the 8-oxolinalyl acetate (5.9 ng/L) but displayed a complete change in the odor quality to reveal *greasy, rancid*, and *musty* attributes rather than the *citrus-like, soapy*, and *lemon-like* qualities of the 8-oxolinalyl acetate (Table 2).

## Conclusion

From the previous results, one can deduce first insights into structure-odor relationships for the investigated linalool derivatives. Amongst others, the presence of a hydroxy group at C-3 in linalool is the main contributor to both odor quality and potency of all mentioned compounds in this study; thereby, the C-8 position does not contain any functionality in case of linalool. On the contrary, the acetate derivative of this hydroxy group, linalyl acetate, displayed low odor potency. However, we could show that this is compensated by C-8 oxidation yielding 8-oxolinalyl acetate and the 8-carboxylinalyl acetate with low thresholds that are in a comparable range as the threshold of linalool but eliciting different odor attributes. On the other hand we could show that the reduced moiety at the C-8 oxidation products yielding the corresponding hydroxy function, does not positively contribute to odor potency, irrespective of whether the C-3 bares a hydroxy or an ester function; this structural modification resulted in the highest odor thresholds determined within this study. To sum up, it can be concluded that in view of the investigated substances predominantly the C-3 substitution with a hydroxy group, a relatively non-voluminous and polar ligand, is important for high odor potency and the characteristic smell properties that are related to linalool. If this hydroxy group is esterified, then C-8 substitution with either an aldehyde or a carboxyl group is crucial to maintain the odor threshold, albeit, thereby losing the specific odor character. Any other structural changes investigated within this study led to either drastic decrease in the potency or even total odor loss.

### Conflict of interest statement

The authors declare that the research was conducted in the absence of any commercial or financial relationships that could be construed as a potential conflict of interest.
